# Pharyngeal phase of swallowing in post-stroke dysphagia: videoendoscopy and speech-language-hearing assessment

**DOI:** 10.1590/2317-1782/20242023242en

**Published:** 2024-08-19

**Authors:** Ramon Cipriano Pacheco de Araújo, Lidiane Maria de Brito Macedo Ferreira, Cynthia Meira de Almeida Godoy, Hipólito Magalhães

**Affiliations:** 1 Programa de Pós-graduação em Fonoaudiologia, Universidade Federal do Rio Grande do Norte – UFRN - Natal (RN), Brasil.; 2 Hospital Universitário Onofre Lopes, Universidade Federal do Rio Grande do Norte – UFRN - Natal (RN), Brasil.; 3 Departamento de Fonoaudiologia, Universidade Federal do Rio Grande do Norte – UFRN - Natal (RN), Brasil.

**Keywords:** Deglutition Disorders, Dysphagia, Stroke, Respiratory Aspiration, Pharynx

## Abstract

**Purpose:**

To investigate the outcomes of fiberoptic endoscopic evaluation of pharyngeal swallowing phase and clinical evaluation of swallowing among dysphagic individuals with and without chronic stroke in different food consistencies.

**Methods:**

This is a cross-sectional and retrospective study based on data collection from medical records. 134 swallowing video endoscopy exams of dysphagic patients were analyzed, in which they were divided into two groups according to the diagnosis of stroke, in which data were collected regarding mobility and strength of the tongue, phonation and cough efficiency, and the pharyngeal signs of dysphagia with four food consistencies from the International Dysphagia Diet Standardization Initiative (IDDSI), for comparison between groups. To analyze and classify the severity of pharyngeal residues, the Yale Pharyngeal Residue Severity Rating Scale (YPRSRS) was used by two independent professionals.

**Results:**

There was a significant difference in the presence of pharyngeal residue, laryngeal penetration and laryngotracheal aspiration in all consistencies evaluated (level 0, 2, 4 and 7) (p= <0.001), in addition to the association with multiple swallowing in thin liquid, slightly thickened liquid and solid (level 0, 2 and 7) (p= 0.026).

**Conclusion:**

Dysphagic individuals diagnosed with stroke showed differences in videoendoscope signs of pharyngeal residue, laryngeal penetration and laryngotracheal aspiration, regardless of the food consistency assessed, compared to dysphagic individuals without the diagnosis. Just as there was a difference in the finding of multiple swallowing only in the consistencies of thin liquid, extremely thickened liquid and solid.

## INTRODUCTION

Stroke is the leading cause of death worldwide^(1)^, with an expected increase in incidence due to the aging of the global population^(1)^. Stroke is categorized into two main types (ischemic and hemorrhagic) and three phases (the acute phase in the first 2 weeks after the injury, the subacute phase up to 6 months, and the chronic phase after 6 months after the injury)^(2)^. The incidence of post-stroke oropharyngeal dysphagia, especially in the first weeks, is 50% of cases^(3)^, which can increase up to 78% depending on the type and severity of the injury and individual comorbidities such as diabetes mellitus^(1)^.

Swallowing is a complex process involving coordination between five cranial nerve pairs and 26 muscle groups. Although healthy individuals perform this task frequently and effortlessly, the motor and sensory components of swallowing may be compromised in some post-stroke patients^(3)^. Thus, some evidence has proposed that the laterality of the affected hemisphere and brain lesions in the anterior insula are associated with greater severity of dysphagia in post-stroke patients so that the insula has important connections with the primary and supplementary motor cortex, medial nucleus of the thalamus, and the nucleus of the solitary tract that participate in the mediation of oropharyngeal swallowing^(4,5)^.

The impairment caused by brain injuries resulting from stroke can affect both the oral and pharyngeal phases of swallowing, depending on the degree of severity. The oral phase involves different processes of mastication, salivation, qualification, organization, and propulsion of the food bolus. Hence, possibly reduced tongue mobility and strength cause incoordination of the food bolus and increased oral transit time, hindering propulsion and its transfer to the subsequent phase^(6)^. As for the pharyngeal phase, its changes occur mainly due to delayed onset time of the pharyngeal response, silent aspirations due to reduced afferent component of the cough mechanism, and increased amounts of residue in pharyngeal recesses after swallowing ^(7, 8)^.

Thus, post-stroke dysphagic patients with compromised swallowing efficiency and safety are at greater risk of aspirating ingested food. Moreover, cases of laryngotracheal aspiration confirmed by instrumental evaluation are at an 11 times greater risk of developing aspiration pneumonia, being hospitalized, and needing a nasogastric tube or gastrostomy to maintain adequate nutritional levels^(9-11)^.

Given the pathophysiology present in all swallowing phases, this study hypothesized that objective and subjective swallowing assessments differ between dysphagic patients with and without a diagnosis of stroke. Therefore, this study aimed to investigate the findings of videoendoscopy and speech-language-hearing (SLH) clinical assessment of dysphagic individuals with and without chronic stroke using different food consistencies.

## METHODS

This cross-sectional retrospective study was based on data collected from medical records. The research was conducted at the otorhinolaryngology outpatient clinic of the Onofre Lopes University Hospital, where data were collected from the medical records of fiberoptic endoscopic evaluation of swallowing (FEES) of patients monitored between 2015 and 2022. All participants or their legal guardians signed a standard informed consent form, made available by the service before carrying out the research procedures. The study was approved by the Research Ethics Committee of the Onofre Lopes University Hospital, under evaluation report no. 5.146.899. The study collected SLH assessment data and FEES findings.

### Sample

The convenience sample of individuals seeking care at the said location included 134 adults. The first group had 44 dysphagic individuals aged 37 to 97 years, with a mean age of 67.45 (±13.9) years, predominantly males, who had a clinical history of chronic ischemic stroke, regardless of the time of diagnosis. The exclusion criteria in this group were individuals with an alternative feeding route, tracheostomy, a history of cancer treatment, other neurological diagnoses, and an inability to follow commands.

The second group had mostly older adults being investigated for dysphagia due to idiopathic causes. It comprised 90 dysphagic individuals aged 52 to 105 years, with a mean age of 61.88 (±15.8) years, predominantly females, who did not have a history of stroke. The exclusion criteria in this group were individuals with an alternative feeding route, tracheostomy, history of cancer treatment, other neurological diagnoses, and inability to follow commands.

Both groups comprised individuals with clinical complaints of oropharyngeal dysphagia screened by health professionals in the service without a standardized protocol and/or referred by other sectors of the hospital, who performed the FEES for objective and instrumental investigation of swallowing.

### Procedures

The clinical evaluation was performed by an SLH pathologist from the service just before the instrumental examination. The preliminary evaluation uses a protocol specific to the service, which analyzes the orofacial myofunctional aspects involved in the oral phase of swallowing. This research analyzed aspects such as tongue strength and mobility, spontaneous coughing on command, and maximum phonation time. Tongue weakness is observed when the evaluator asks the patient to use maximum voluntary tongue strength against the resistance of the evaluator's gloved finger, resulting in a short-lasting muscle contraction and a rapid decrease in isometric movement – although this is a qualitative measure depending on previous experience in comparison with normality. The SLH evaluator also asked the patient to perform tongue protrusion and lateralization and protrusion against the resistance of a gloved finger, produce a strong spontaneous cough, and emit the prolonged sound of the vowel /a/ for as long as possible, after demonstrating a model. The following parameters and criteria of normality were used: the ability to correctly execute the desired commands, maintain isometric force on the finger, subjective efficiency in cough production (efficient/weak) for possible pharyngeal clearance, and a maximum phonation time of 14 seconds for women and 20 seconds for men. All changes were recorded; in case of doubt, they repeated the procedures before continuing.

The FEES was performed by a resident physician, the head otorhinolaryngologist, and an SLH pathologist with experience in oropharyngeal dysphagia, in accordance with the institution's protocol. A flexible Olympus^®^ nasofibrolaryngoscope measuring 3.2 mm in diameter, with a micro-camera and light source, was inserted into the nasal cavity up to the hypopharynx. The patient was instructed to remain seated throughout the exam, and no topical anesthetic was used. During the exam, the SLH pathologist offered food in different consistencies artificially colored with aniline blue, in the following order: level 2 (mildly thick liquid), level 4 (extremely thick liquid), level 0 (thin liquid), and level 7 (regular solid food), according to the International Dysphagia Diet Standardization Initiative (IDDSI) classification^(12)^, three times in a 5 ml metal spoon. The liquids were artificially flavored diet juice thickened with an instant cornstarch food thickener, while the solid food consisted of 8 g crackers.

The three professionals mentioned above, with experience in the examination, interpreted, simultaneously evaluated by consensus, and concluded whether there were multiple swallows, posterior oral spillage, pharyngeal residue in the valleculae and/or pyriform sinuses according to the Yale Pharyngeal Residue Severity Rating Scale (YPRSRS)^(13)^ (1 - None, 2 - Pharyngeal residue, 3 - Mild residue, 4 - Moderate residue, 5 - Severe residue), laryngeal penetration, and laryngotracheal aspiration. The residue was classified according to the highest occurrence of severity after the three swallows of each food consistency. The analysis considered the following parameters, from the first offer onwards: multiple swallows: more than two attempts to swallow the same offer; posterior oral spillage: premature food spillage in the hypopharynx before initiating swallowing; pharyngeal residue: residual presence of colored food in the valleculae and/or pyriform sinuses from the first offering; laryngeal penetration: residual presence of colored food in the vocal folds; and laryngotracheal aspiration: residual passage of colored food below the vocal folds. All analyses were performed in real time, and the images were stored on a computer at the clinic to be reviewed as many times as the professionals deemed necessary after the examination.

The data underwent descriptive and inferential statistical analyses, using measures of central tendency, proportions, and frequencies. The Kolmogorov-Smirnov verified the normal distribution of quantitative variables. Pearson’s chi-square test or Fisher’s exact test were applied for the variables “SLH assessment” and “videoendoscopy pharyngeal signs,” depending on whether the expected frequency for each cell was greater than or equal to 5. Residue severity was dichotomized into “trace to mild residue” (YPRSRS 2-3) and “moderate to severe residue” (YPRSRS 4-5) for intragroup comparison, at the 0.05 significance level.

## RESULTS

A total of 134 FEES were analyzed, stratifying the sample into two groups according to the diagnosis of stroke: 44 dysphagic individuals with stroke and 90 dysphagic individuals without the diagnosis. Table 1 shows the distribution of variables and the comparison of the findings of the previous SLH assessment between the groups. There was a predominance of males, reduced tongue mobility and strength, and reduced maximum phonation time in the group of dysphagic individuals with stroke than in the one without stroke.

**Table 1:** Distribution of variables and comparison of speech-language-hearing findings between groups

	Groups		
**Variables**	**With stroke**	**Without stroke**	***p*-value**
Age (years)*	67.45 (±13.9)	61.88 (±15.8)	
Sex n(%)			
Males	28 (63.3)	28 (31.1)	
Females	16 (36.4)	62 (68.9)	
**Speech-language-hearing assessment n(%)**			
Tongue strength			
Adequate	25 (56.8)	79 (87.7)	<0.001**
Reduced	19 (43.2)	11 (12.3)	
Tongue mobility			
Preserved	23 (52.2)	71 (78.9)	0.001**
Abnormal	21 (47.8)	19 (21.1)	
Spontaneous cough			
Efficient	31 (70.4)	75 (83.3)	0.086
Weak	13 (29.6)	15 (16.7)	
Maximum phonation time			
Adequate	18 (40.9)	53 (58.9)	**0.051** **
Reduced	26 (59.1)	37 (41.1)	

* values shown in means and standard deviations;

** Pearson’s chi-square test

**Caption:** n(%) = absolute and relative frequency

Table 2 presents the FEES findings regarding multiple swallows, posterior oral spillage, pharyngeal residues, laryngeal penetration, and laryngotracheal aspiration in different food consistencies between the groups. The results suggest that the group of dysphagic individuals with stroke had pharyngeal residues, laryngeal penetration, and laryngotracheal aspiration more often in all food consistencies evaluated than the one without stroke. There was a significant difference in multiple swallows of three consistencies, namely: thin liquid (level 0), extremely thick liquid (level 4), and regular solid food (level 7). On the other hand, there was no difference in posterior oral spillage between the groups in any of the consistencies. The group with stroke was the only one with laryngotracheal aspirations of thick liquids and solid food.

**Table 2:** Association of pharyngeal signals with different food consistencies between groups

	Groups		
**Pharyngeal signals per food consistency level (IDDSI)**	**With stroke**	**Without stroke**	***p*-value**
	**n = 44 (%)**	**n = 90 (%)**	
Thin liquid (level 0)			
Multiple swallows			
Yes	5 (11.3)	2 (2.2)	0.026**
No	39 (88.6)	88 (97.7)	
Posterior oral spillage			
Yes	25 (56.8)	39 (43.3)	0.142*
No	19 (43.1)	51 (56.6)	
Pharyngeal residue			
Yes	18 (40.9)	11 (12.2)	<0.001*
No	26 (59.0)	79 (87.7)	
Laryngeal penetration			
Yes	19 (43.1)	7 (7.7)	<0.001*
No	25 (56.8)	83 (92.2)	
Laryngotracheal aspiration			
Yes	11 (25.0)	3 (3.3)	<0.001**
No	33 (75.0)	87 (96.6)	
Mildly thick liquid (level 2)			
Multiple swallows			
Yes	5 (11.3)	3 (3.3)	0.065**
No	39 (88.6)	87 (96.6)	
Posterior oral spillage			
Yes	18 (40.9)	30 (33.3)	0.390*
No	26 (59.0)	60 (66.6)	
Pharyngeal residue			
Yes	21 (47.7)	11 (12.2)	<0.001*
No	23 (52.2)	79 (87.7)	
Laryngeal penetration			
Yes	12 (27.2)	2 (2.2)	<0.001**
No	32 (72.7)	88 (97.7)	
Laryngotracheal aspiration			
Yes	6 (13.6)	0 (0.0)	<0.001**
No	38 (86.3)	90 (100)	
Extremely thick liquid (level 4)			
Multiple swallows			
Yes	5 (11.3)	2 (2.2)	0.026**
No	39 (88.6)	88 (97.7)	
Posterior oral spillage			
Yes	19 (43.1)	26 (28.8)	0.100*
No	25 (56.8)	64 (71.1)	
Pharyngeal residue			
Yes	23 (52.2)	9 (10.0)	<0.001*
No	21 (47.7)	81 (90.0)	
Laryngeal penetration			
Yes	12 (27.2)	2 (2.2)	<0.001**
No	32 (72.7)	88 (97.7)	
Laryngotracheal aspiration			
Yes	7 (15.9)	0 (0.0)	<0.001**
No	37 (84.0)	90 (100)	
Regular solid food (level 7)			
Multiple swallows			
Yes	6 (13.6)	2 (2.2)	0.009**
No	38 (86.3)	88 (97.7)	
Posterior oral spillage			
Yes	16 (36.3)	24 (26.6)	0.249*
No	28 (63.6)	66 (73.3)	
Pharyngeal residue			
Yes	17 (38.6)	8 (8.8)	**<0.001** *
No	27 (61.3)	82 (91.1)	
Laryngeal penetration			
Yes	7 (15.9)	1 (1.1)	0.001**
No	37 (84.0)	89 (98.8)	
Laryngotracheal aspiration			
Yes	4 (9.0)	0 (0.0)	**0.004** **
No	40 (90.9)	90 (100)	

* Pearson’s chi-square test;

** Fisher’s exact test

**Caption:** n(%) = absolute and relative frequency; IDDSI = International Dysphagia Diet Standardisation Initiative

Table 3 shows the severity of pharyngeal residue between groups. The intragroup analysis shows that moderate to severe residue (YPRSRS 4-5) occurred more often in the group of dysphagic individuals with stroke, while the one without stroke had trace to mild pharyngeal residues (YPRSRS 2-3) more often in all food consistencies. This difference was significant with thin liquids (level 0) and mildly thick liquids (level 2) in the group with stroke.

**Table 3:** Association between the severity of pharyngeal residues of different food consistencies between groups

	With stroke			Without stroke		
	YPRSRS			YPRSRS		
**Residue severity per consistency level (IDDSI)**	**Trace to mild residue**	**Moderate to severe residue**	***p*-value**	**Trace to mild residue**	**Moderate to severe residue**	***p*-value**
Thin liquid (level 0)						
Yes	5 (11.3)	13 (29.5)	**0.050**	7 (7.7)	4 (4.4)	**0.004**
No	39 (88.6)	31 (70.4)		83 (92.2)	86 (95.5)	
Mildly thick liquid (level 2)						
Yes	6 (13.6)	15 (34.0)	**0.032**	8 (8.8)	3 (3.3)	0.069
No	38 (86.3)	29 (65.9)		82 (91.1)	87 (96.6)	
Extremely thick liquid (level 4)						
Yes	9 (20.4)	14 (31.8)	0.378	6 (6.6)	3 (3.3)	**0.025**
No	35 (79.5)	30 (68.1)		84 (93.3)	87 (96.6)	
Regular solid food (level 7)						
Yes	5 (11.3)	12 (27.2)	0.090	5 (5.5)	3 (3.3)	**0.013**
No	39 (88.6)	32 (72.7)		85 (94.4)	87 (96.6)	

**Caption:** IDDSI = International Dysphagia Diet Standardisation Initiative; YPRSRS = Yale Pharyngeal Residue Severity Rating Scale; Pearson’s chi-square test

## DISCUSSION

The study verified that pharyngeal residues, laryngeal penetration, and laryngotracheal aspiration were significantly different, regardless of the consistency, between dysphagic individuals with and without stroke. The group with stroke had moderate to severe pharyngeal residues (YPRSRS 4-5) more often than the other group, which had trace to mild residues (YPRSRS 2-3) more often.

Individuals with neurogenic oropharyngeal dysphagia are known to have pharyngeal residues after swallowing, as this condition is related to neuromuscular disorders that affect swallowing biomechanical efficiency^(10-12)^. However, few studies have compared the presence and severity of pharyngeal residues of different food consistencies between these patients and other dysphagic individuals without the diagnosis^(14-16)^. This information is important to understand these patients’ pathophysiology and the process of rehabilitating the swallowing function, given the existing evidence that pharyngeal residue in post-stroke dysphagia is predictive of recovery and persistent throughout this process^(17)^.

Tongue strength and mobility, verified in the previous SLH assessment (even if subjectively), are essential to understanding the process of formation and transport of the food bolus in the oral cavity. There is evidence that lesions resulting from stroke often result in tongue weakness and incoordination and certain relationships with impaired aspects of oral processing^(18-20)^. Our study results corroborate the premise of tongue weakness since the group of dysphagic patients with stroke had significantly different tongue mobility and strength (mostly reduced) from the other group. Thus, it is understood that post-stroke dysphagic patients develop greater impairments in the anteroposterior movements necessary for transporting the food bolus in the oral cavity, as well as decreased strength to propel this food down to the pharyngeal phase^(21,22)^. This occurs because the intrinsic tongue muscles have reduced amplitude of motor potential intervals, evidenced by the electrophysiological approach^(23)^, resulting in decreased tongue movements.

The pharyngeal residue was an important finding in the study, given its significantly different occurrence and severity in all food consistencies between the groups. The group with stroke had a higher occurrence of moderate to severe residue of extremely thick liquids (level 4), which corroborates the understanding that these patients perform inefficient swallowing. Nevertheless, studies still diverge on the residue severity classification in this population, since there is evidence reporting mild residues^(14,24)^, while other studies report moderate to severe residues^(25,26)^. This difference is based mainly on the individual characteristics of the patient’s phase of the disease and the location of the brain injury. There is evidence that lesions in specific areas of the supramarginal gyrus, angular gyrus, postcentral gyrus, and parietotemporal cortical regions are associated with a greater presence of pharyngeal residue^(26)^, indicating areas involved in these individuals’ severity of dysphagia.

The pharyngeal strength is another aspect described in the literature related to pharyngeal residues in this population. It is triggered by the hyolingual complex of elevation and opening of the upper esophageal sphincter, described as a preponderant factor in the occurrence of pharyngeal residues in valleculae and/or pyriform sinuses^(27)^. Even if the upper esophageal sphincter opening was inefficient, this difficulty could be overcome by the pressure of food bolus propulsion exerted by the tongue and hyolaryngeal excursion^(28)^.

Oral spillage was a videoendoscopy finding with no significant difference between the groups, although the one with stroke had approximately 56.8% of occurrences with thin liquid (level 0). This characteristic is little described in research that evaluates post-stroke swallowing, but it is a consequence of the mechanisms of pharyngeal response delay and difficulties in tongue mobility and strength to contain the bolus in the oral cavity, evidenced in the SLH assessment.

The results of laryngeal penetration and laryngotracheal aspiration showed a significant difference between the groups, regardless of the consistency evaluated. Patients with post-stroke dysphagia had such occurrences more often, compromising their swallowing safety mechanism^(29)^. The instrumental evaluation presents evidence that this finding is directly related to the severity of the residues after swallowing – more severe residues, whether in valleculae or pyriform sinuses, contribute to greater chances of laryngotracheal penetration and aspiration in this population, due to the deficit in expelling this material from the larynx^(30)^.

Coughing on command was one of the subjective parameters assessed before the instrumental examination in this study – in which there was no difference between the groups. Although no association was found, this parameter must be investigated, since some cases of stroke may have overlapping lesions associated with weak coughing and even decreased pharyngeal sensitivity, which worsens the prognosis of oropharyngeal dysphagia^(31)^. Although the mechanisms of swallowing and coughing are independent, both are complex and coordinated motor actions that reconfigure the individual's laryngeal pattern^(31)^.

Maximum phonation time is a reliable vocal assessment measure related to multimodal perceptual and objective measures of dysphonia severity and, consequently, dysphagia severity^(32,33)^. Our study found a significant difference between the groups, indicating that 59.1% of post-stroke dysphagic individuals in the chronic phase had reduced maximum phonation time in relation to their sex, which suggests reduced glottal closure during phonation. As it is a quick and easy parameter to collect in clinical practice, it provides the SLH pathologist with important information regarding possible speech changes and lower airway protection during swallowing, Moreover, this parameter is little researched in dysphagic patients who apparently do not have other speech changes^(31)^. Since pharyngeal residues are frequent, glottal closure and the cough reflex must be rehabilitated to efficiently trigger the lower airway protection reaction and pharyngeal cleaning to avoid the silent aspirations that are common in post-stroke patients^(26)^.

The limitations of the study include its type, which did not allow us to verify the cause and effect or idiopathic cause of dysphagia in the group without stroke or differentiate the physiological mechanism of the lesion – i.e., the topographic location that prevailed in the group with stroke. This definition is knowingly important to determine the prognosis, recurrence, and treatment with specific measures. However, all patients had a diagnosis of ischemic stroke in the chronic phase of the disease. The results of this study provide an objective and subjective characterization of the pathophysiology of the oral and pharyngeal phases in post-stroke dysphagic patients in comparison with dysphagic patients without this impairment. Hence, they raise new hypotheses regarding the effects of new therapeutic programs in SLH rehabilitation.

## CONCLUSION

Dysphagic individuals diagnosed with stroke had differences in videoendoscopic signs of pharyngeal residues, laryngeal penetration, and laryngotracheal aspiration, regardless of the food consistency assessed, from dysphagic individuals without the diagnosis. There was also a difference in findings of multiple swallowing only with thin liquid, extremely thick liquid, and solid food.
