# Supplementary material for: Pharyngeal phase of swallowing in post-stroke dysphagia: videoendoscopy and speech-language-hearing assessment
Source: Codas. 2024 Aug 19;36(5):e20230242. doi: 10.1590/2317-1782/20242023242en (PMC11340873; doi:10.1590/2317-1782/20242023242en)
Supplement: Translation

# Translation: Fase faríngea da deglutição na disfagia pós-AVE: achados videoendoscópios e da avaliação fonoaudiológica

**DOI:** 10.1590/2317-1782/20242023242pt

**Authors:** Ramon Cipriano Pacheco de Araújo, Lidiane Maria de Brito Macedo Ferreira, Cynthia Meira de Almeida Godoy, Hipólito Magalhães

**Affiliations:** 1 Programa de Pós-graduação em Fonoaudiologia, Universidade Federal do Rio Grande do Norte – UFRN - Natal (RN), Brasil.; 2 Hospital Universitário Onofre Lopes, Universidade Federal do Rio Grande do Norte – UFRN - Natal (RN), Brasil.; 3 Departamento de Fonoaudiologia, Universidade Federal do Rio Grande do Norte – UFRN - Natal (RN), Brasil.

**Keywords:** Transtornos de Deglutição, Disfagia, Acidente Vascular Encefálico, Aspiração Respiratória, Faringe

## Abstract

**Objetivo:**

Investigar os achados videoendoscópios da fase faríngea da deglutição e da avaliação clínica da deglutição, entre indivíduos disfágicos com e sem AVE crônico em diferentes consistências alimentares.

**Método:**

Trata-se de um estudo transversal e retrospectivo com base na coleta de dados dos prontuários. Foram analisados 134 exames da videoendoscopia da deglutição de pacientes disfágicos, em que foram divididos em dois grupos de acordo com o diagnóstico de AVE, na qual, foram coletados dados com relação à mobilidade e força de língua, fonação e eficiência da tosse, e os sinais faríngeos de disfagia com quatro consistências alimentares do International Dysphagia Diet Standartisation Initiative (IDDSI), para comparação entre os grupos. Para análise e classificação da gravidade dos resíduos faríngeos, foi utilizado o Yale Pharyngeal Residue Severity Rating Scale (YPRSRS) por dois profissionais independentes.

**Resultados:**

Houve diferença significativa com a presença de resíduos faríngeos, penetração laríngea e aspiração laringotraqueal em todas as consistências avaliadas (nível 0, 2, 4 e 7) (p= <0,001), além da associação com deglutições múltiplas em líquido ralo, líquido levemente espessado e sólido (nível 0, 2 e 7) (p= 0,026).

**Conclusão:**

Os indivíduos disfágicos com diagnóstico de acidente vascular encefálico apresentaram diferença nos sinais videoendoscópios de resíduos faríngeos, penetração laríngea e aspiração laringotraqueal independentemente da consistência alimentar avaliada, em comparação aos indivíduos disfágicos sem o diagnóstico. Assim como houve diferença no achado da deglutição múltipla apenas nas consistências de líquido ralo, líquido extremamente espessado e sólido.

## INTRODUÇÃO

O acidente vascular encefálico (AVE) é a principal causa de morte em todo mundo^(1)^, com previsão de aumento de sua incidência devido a tendência de envelhecimento da população mundial^(1)^. O AVE é categorizado em dois tipos principais, sendo o isquêmico e o hemorrágico, com três fases, denominado fase aguda nas duas primeiras semanas da lesão, fase subaguda até seis meses e fase crônica após seis meses da lesão^(2)^. A incidência da disfagia orofaríngea após o AVE, principalmente nas primeiras semanas, é de 50% dos casos^(3)^, o que pode aumentar em até 78% a depender do tipo e da gravidade da lesão, além de algumas comorbidades individuais como a diabetes mellitus^(1)^.

A deglutição é um processo complexo que envolve a coordenação entre cinco pares de nervos cranianos e vinte e seis grupos musculares que, embora essa tarefa ocorra com frequência e sem esforço entre indivíduos saudáveis, os componentes motores e sensoriais da deglutição podem estar comprometidos em alguns pacientes pós-AVE^(3)^. Dessa forma, algumas evidências propuseram que a lateralidade do hemisfério afetado e as lesões cerebrais na região de ínsula anterior estão associadas à maior severidade da disfagia no pós-AVE, de modo que, a região de ínsula possui conexões importantes com o córtex motor primário e suplementares, núcleo medial do tálamo e o núcleo do trato solitário que participam na mediação da deglutição orofaríngea^(4,5)^.

O comprometimento provocado pelas lesões cerebrais advindas do AVE podem afetar tanto a fase oral quanto a fase faríngea da deglutição, a depender do grau de severidade. A fase oral envolve diferentes processos de mastigação, insalivação, qualificação, organização e propulsão do bolo alimentar que, a partir de eventuais mudanças na redução de mobilidade e força de língua, provocam incoordenação do bolo alimentar e tempo de trânsito oral aumentado, que dificultam a propulsão e sua transferência para a fase conseguinte^(6)^. Enquanto que, na fase faríngea, suas alterações concentram-se no atraso no tempo de início de resposta faríngea, aspirações silenciosas devido a redução no componente aferente do mecanismo de tosse e no aumento dos resíduos em recessos faríngeos após a deglutição^(7,8)^.

Nesse contexto, com a eficiência e a segurança da deglutição comprometidas, os pacientes disfágicos pós-AVE possuem riscos maiores de aspirar o alimento ingerido, que por consequência, quando confirmado a aspiração laringotraqueal pela avaliação instrumental, o risco aumenta 11 vezes mais de desenvolver pneumonia aspirativa, ser hospitalizado e necessitar da administração de uso de sonda nasogástrica ou gastrostomia para manter os níveis nutricionais adequados^(9-11)^.

Dessa forma, ao considerar a fisiopatologia presente em todas as fases da deglutição, a hipótese levantada é que haverá diferenças na avaliação objetiva e subjetiva da deglutição entre pacientes disfágicos com e sem o diagnóstico de AVE. Portanto, o objetivo deste estudo foi investigar os achados videoendoscópios e fonoaudiológicos através da avaliação clínica, entre indivíduos disfágicos com e sem AVE crônico em diferentes consistências alimentares.

## MÉTODOS

Trata-se de um estudo transversal e retrospectivo com base na coleta de dados dos prontuários. A pesquisa foi conduzida no ambulatório de otorrinolaringologia do Hospital Universitário Onofre Lopes, em que foram coletados dados nos prontuários do exame da videoendoscopia da deglutição (VED) dos pacientes acompanhados entre os anos de 2015 a 2022. Todos os participantes, ou representantes legais, assinaram o Termo de Consentimento Livre e Esclarecido (TCLE) padrão disponibilizado pelo serviço antes da realização dos procedimentos da pesquisa. O estudo foi aprovado pelo Comitê de Ética em Pesquisa do Hospital Universitário Onofre Lopes, sob parecer n° 5.146.899. Foram coletados dados referentes à avaliação clínica fonoaudiológica e aos achados na VED.

### Casuística

A amostra escolhida por conveniência entre indivíduos que buscaram atendimento nesse local foi composta por 134 participantes adultos. O primeiro grupo foi constituído por 44 indivíduos disfágicos com idade entre 37 e 97 anos, média de 67,45 (±13,9) anos e com predomínio do gênero masculino, que apresentavam histórico clínico de acidente vascular encefálico isquêmico em fase crônica, independente do tempo do diagnóstico. Os critérios de exclusão nesse grupo foram indivíduos em uso de via alternativa de alimentação, usuários de traqueostomia, histórico de tratamento oncológico, outros diagnósticos neurológicos e incapazes de seguir comandos.

Enquanto que no segundo grupo, foram incluídos indivíduos em investigação da disfagia por causa idiopática e em sua maioria adultos mais velhos. Assim, o grupo foi constituído por 90 indivíduos disfágicos com idade entre 52 a 105 anos, média de 61,88 (±15,8) anos e com predomínio do gênero feminino, que não apresentavam o histórico de AVE. Os critérios de exclusão nesse grupo foram indivíduos em uso de via alternativa de alimentação, usuários de traqueostomia, histórico de tratamento oncológico, outros diagnósticos neurológicos e incapazes de seguir comandos.

Ambos os grupos compostos por indivíduos com queixas clínicas de disfagia orofaríngea rastreados por profissionais de saúde do serviço sem um protocolo padronizado e/ou encaminhados por outros setores do próprio hospital, que realizaram o exame da VED para investigação objetiva e instrumental da deglutição.

### Procedimentos

A avaliação clínica foi realizada por um fonoaudiólogo do serviço momentos antes da realização do exame instrumental. Na avaliação prévia é utilizado um protocolo próprio do serviço, na qual, é analisado aspectos miofuncionais orofaciais envolvidos na fase oral da deglutição. Para esta pesquisa foram analisados aspectos como: força e mobilidade de língua, tosse espontânea sob comando e o tempo máximo de fonação. A fraqueza lingual é observada quando o avaliador solicita que o paciente utilize a força máxima voluntária de língua contra a resistência do dedo enluvado do próprio avaliador, que apesar de ser medidas qualitativas a depender da experiência prévia de comparação da normalidade, resulta em uma baixa duração de contração muscular e diminuição rápida do movimento isométrico. Além disso, o avaliador fonoaudiólogo foi responsável por solicitar ao paciente que executasse os movimentos desejados de protrusão e lateralização com a língua, protrusão contra a resistência do dedo enluvado, produzisse uma tosse espontânea forte e emitisse o som prolongado da vogal /a/ o máximo de tempo possível, após demonstração de modelo. Foi adotado como parâmetros e critérios de normalidade a capacidade de executar corretamente os comandos desejados, manter força isométrica sobre o dedo, a eficiência subjetiva na produção da tosse (eficiente/fraca) para uma eventual limpeza faríngea e o tempo máximo de fonação cronometrado de 14s para as mulheres e 20s para homens. Todas as alterações são devidamente anotadas, e em caso de dúvida, repetida para dar prosseguimento.

O exame da videoendoscopia da deglutição é realizado por um médico residente, um otorrinolaringologista responsável e pelo fonoaudiólogo com experiência em disfagia orofaríngea, em consonância com o protocolo da instituição. É utilizado uma nasofibrolaringoscópio flexível, marca Olympus^®^ de 3,2mm de diâmetro, com microcâmera e fonte de luz, introduzida na cavidade nasal até a região de hipofaringe. O paciente é orientado que permaneça na posição sentado durante toda realização do exame, não sendo utilizado anestésico tópico.

Ainda durante a realização do exame, o fonoaudiólogo ofertada alimentos corados artificialmente com anilina azul em diferentes consistências alimentares, seguindo esta ordem: nível 2 (líquido levemente espessado), nível 4 (líquido extremamente espessado), nível 0 (líquido ralo) e nível 7 (sólido normal), de acordo com a classificação *International Dysphagia Diet Standardisation* *Initiative* (IDDSI)^(12)^, com três ofertas em uma colher metálica de 5ml. Os líquidos foram preparados em forma de suco dietético saborizado artificialmente e espessado por um espessante alimentar instantâneo de amido de milho, enquanto o alimento sólido foi constituído por uma porção de biscoito salgado de 8g.

Os três profissionais anteriormente citados, com experiência na realização do exame, foram responsáveis por interpretar, avaliar simultaneamente, por consenso e concluíram se havia presença de deglutições múltiplas, escape oral posterior, resíduos faríngeos em região de valéculas e/ou seios piriformes de acordo com a escala *Yale Pharyngeal Residue Severity Rating Scale* (YPRSRS)^(13)^ (1 - Nenhum, 2 - Vestígio faríngeo, 3 - Resíduo leve, 4 - Resíduo moderado, 5 - Resíduo grave), além da presença de penetração laríngea e aspiração laringotraqueal. O resíduo foi classificado na maior ocorrência de gravidade após as três deglutições de cada consistência alimentar. Foram considerados para a análise, a partir da primeira oferta, os seguintes parâmetros: deglutições múltiplas a presença de mais de duas tentativas de deglutir a mesma oferta; escape oral posterior a presença do escape prematuro do alimento em região de hipofaringe antes de desencadear a deglutição; resíduo faríngeo a presença residual de alimento corada em região de valéculas e/ou seios piriformes a partir da primeira oferta; penetração laríngea a presença residual de alimento corado em região de pregas vocais; e aspiração laringotraqueal a passagem residual de alimento corado abaixo das pregas vocais. Toda análise aconteceu em tempo real e as imagens foram armazenadas em um computador do próprio ambulatório, para serem revisadas quantas vezes os profissionais julgassem necessárias após a realização do exame.

Para análise dos dados, foi utilizada a estatística descritiva e inferencial por meio de medidas de tendência central, proporções e frequências. Para análise das variáveis do estudo, foi utilizado o teste Kolmogorov-Smirnov para verificar a distribuição normal nas variáveis quantitativas, de modo que, para as variáveis “avaliação fonoaudiológica” e “sinais faríngeos videoendoscópios”, aplicou-se o teste Qui-quadrado de Pearson ou teste Exato de Fisher, a depender da frequência esperada por cada célula ser superior ou igual a 5. A gravidade dos resíduos foi dicotomizada em “vestígio a resíduo leve” (YPRSRS 2-3) e “resíduo moderado a grave” (YPRSRS 4-5) para comparação intragrupos, em nível de significância de 0,05.

## RESULTADOS

Foram analisados 134 exames da VED, onde a amostra foi estratificada em dois grupos de acordo com o diagnóstico de AVE. Sendo, 44 indivíduos disfágicos com AVE e 90 indivíduos disfágicos sem o diagnóstico. Na Tabela 1 está representada a distribuição das variáveis e a comparação dos achados da avaliação fonoaudiológica prévia entre os grupos. Houve predomínio do gênero masculino, mobilidade de língua reduzida, força de língua reduzida e o tempo máximo de fonação reduzido no grupo de disfágicos com AVE em comparação ao grupo sem AVE.

**Tabela 1:** Distribuição das variáveis e comparação dos achados fonoaudiológicos entre os grupos

Grupos			
**Variáveis**	**Com AVE**	**Sem AVE**	**Valor - *p***
Idade (anos)*	67,45 (±13,9)	61,88 (±15,8)	
Gênero n(%)			
Masculino	28 (63,3)	28 (31,1)	
Feminino	16 (36,4)	62 (68,9)	
**Avaliação fonoaudiológica n(%)**			
Força de língua			
Adequada	25 (56,8)	79 (87,7)	<0,001**
Reduzida	19 (43,2)	11 (12,3)	
Mobilidade de língua			
Preservada	23 (52,2)	71 (78,9)	0,001**
Alterada	21 (47,8)	19 (21,1)	
Tosse espontânea			
Eficiente	31 (70,4)	75 (83,3)	0,086
Fraca	13 (29,6)	15 (16,7)	
Tempo máximo de fonação			
Adequado	18 (40,9)	53 (58,9)	**0,051** **
Reduzido	26 (59,1)	37 (41,1)	

* valor expresso em média e desvio padrão;

** Qui-quadrado de Pearson

**Legenda:** n(%) = frequência absoluta e relativa; AVE = acidente vascular encefálico

A Tabela 2 apresenta os achados do exame da VED, com relação à ocorrência de deglutições múltiplas, escape oral posterior, resíduos faríngeos, penetração laríngea e aspiração laringotraqueal, em diferentes consistências alimentares entre os grupos. Os resultados sugerem que o grupo de disfágicos com AVE apresentou maior ocorrência de resíduos faríngeos, penetração laríngea e aspiração laringotraqueal em todas as consistências alimentares avaliadas, em comparação ao grupo de disfágicos sem AVE. Com relação à deglutições múltiplas, houve diferença significativa em três consistências, sendo elas em líquido ralo (nível 0), líquido extremamente espessado (nível 4) e sólido normal (nível 7). Enquanto que, não houve diferença com relação ao escape oral posterior entre os grupos em todas as consistências avaliadas. Nota-se que o grupo com AVE foi o único que apresentou ocorrências de aspiração laringotraqueal em líquidos espessados e sólido.

**Tabela 2:** Associação dos sinais faríngeos em diferentes consistências alimentares entre os grupos

	Grupos		
**Sinais faríngeos por nível de consistência alimentar (IDDSI)**	**Com AVE**	**Sem AVE**	**Valor - *p***
	n = 44 (%)	n = 90 (%)	
Líquido ralo (nível 0)			
Deglutições múltiplas			
Sim	5 (11,3)	2 (2,2)	0,026**
Não	39 (88,6)	88 (97,7)	
Escape oral posterior			
Sim	25 (56,8)	39 (43,3)	0,142*
Não	19 (43,1)	51 (56,6)	
Resíduo faríngeo			
Sim	18 (40,9)	11 (12,2)	<0,001*
Não	26 (59,0)	79 (87,7)	
Penetração laríngea			
Sim	19 (43,1)	7 (7,7)	<0,001*
Não	25 (56,8)	83 (92,2)	
Aspiração laringotraqueal			
Sim	11 (25,0)	3 (3,3)	<0,001**
Não	33 (75,0)	87 (96,6)	
Líquido levemente espessado (nível 2)			
Deglutições múltiplas			
Sim	5 (11,3)	3 (3,3)	0,065**
Não	39 (88,6)	87 (96,6)	
Escape oral posterior			
Sim	18 (40,9)	30 (33,3)	0,390*
Não	26 (59,0)	60 (66,6)	
Resíduo faríngeo			
Sim	21 (47,7)	11 (12,2)	<0,001*
Não	23 (52,2)	79 (87,7)	
Penetração laríngea			
Sim	12 (27,2)	2 (2,2)	<0,001**
Não	32 (72,7)	88 (97,7)	
Aspiração laringotraqueal			
Sim	6 (13,6)	0 (0,0)	<0,001**
Não	38 (86,3)	90 (100)	
Líquido extremamente espessado (nível 4)			
Deglutições múltiplas			
Sim	5 (11,3)	2 (2,2)	0,026**
Não	39 (88,6)	88 (97,7)	
Escape oral posterior			
Sim	19 (43,1)	26 (28,8)	0,100*
Não	25 (56,8)	64 (71,1)	
Resíduo faríngeo			
Sim	23 (52,2)	9 (10,0)	<0,001*
Não	21 (47,7)	81 (90,0)	
Penetração laríngea			
Sim	12 (27,2)	2 (2,2)	<0,001**
Não	32 (72,7)	88 (97,7)	
Aspiração laringotraqueal			
Sim	7 (15,9)	0 (0,0)	<0,001**
Não	37 (84,0)	90 (100)	
Sólido normal (nível 7)			
Deglutições múltiplas			
Sim	6 (13,6)	2 (2,2)	0,009**
Não	38 (86,3)	88 (97,7)	
Escape oral posterior			
Sim	16 (36,3)	24 (26,6)	0,249*
Não	28 (63,6)	66 (73,3)	
Resíduo faríngeo			
Sim	17 (38,6)	8 (8,8)	<0,001*
Não	27 (61,3)	82 (91,1)	
Penetração laríngea			
Sim	7 (15,9)	1 (1,1)	0,001**
Não	37 (84,0)	89 (98,8)	
Aspiração laringotraqueal			
Sim	4 (9,0)	0 (0,0)	0,004**
Não	40 (90,9)	90 (100)	

* Qui-quadrado de Pearson;

** Exato de Fisher

**Legenda:** n(%) = frequência absoluta; AVE = acidente vascular encefálico; IDDSI = International Dysphagia Diet Standardisation Initiative

A Tabela 3 apresenta a gravidade das ocorrências dos resíduos faríngeos entre os grupos. Na análise intragrupo, houve mais ocorrências da gravidade de resíduos moderado a grave (YPRSRS 4-5) no grupo de indivíduos disfágicos com AVE, enquanto que, no grupo de disfágicos sem AVE, houve mais ocorrências da gravidade de vestígio faríngeo a resíduo leve (YPRSRS 2-3) em todas as consistências alimentares avaliadas. Essa diferença foi significativa nas consistências de líquido ralo (nível 0) e líquido levemente espessado (nível 2) no grupo com AVE.

**Tabela 3:** Associação entre a gravidade dos resíduos faríngeos em diferentes consistências alimentares entre os grupos

	**Com AVE**			**Sem AVE**		
	**YPRSRS**			**YPRSRS**		
**Gravidade dos resíduos por nível de consistência (IDDSI)**	**Vestígios a Resíduo leve**	**Resíduo moderado a Grave**	**Valor - *p***	**Vestígios a Resíduo leve**	**Resíduo moderado a Grave**	**Valor - *p***
Líquido ralo (nível 0)						
Sim	5 (11,3)	13 (29,5)	**0.050**	7 (7,7)	4 (4,4)	**0,004**
Não	39 (88,6)	31 (70,4)		83 (92,2)	86 (95,5)	
Líquido levemente espessado (nível 2)						
Sim	6 (13,6)	15 (34,0)	**0,032**	8 (8,8)	3 (3,3)	0,069
Não	38 (86,3)	29 (65,9)		82 (91,1)	87 (96,6)	
Líquido extremamente espessado (nível 4)						
Sim	9 (20,4)	14 (31,8)	0,378	6 (6,6)	3 (3,3)	**0,025**
Não	35 (79,5)	30 (68,1)		84 (93,3)	87 (96,6)	
Sólido normal (nível 7)						
Sim	5 (11,3)	12 (27,2)	0,09	5 (5,5)	3 (3,3)	**0,013**
Não	39 (88,6)	32 (72,7)		85 (94,4)	87 (96,6)	

**Legenda:** AVE = acidente vascular encefálico; IDDSI = International Dysphagia Diet Standardisation Initiative; YPRSRS = Yale Pharyngeal Residue Severity Rating Scale; Qui-quadrado de Pearson

## DISCUSSÃO

Foi possível verificar que indivíduos disfágicos com diagnóstico de AVE apresentaram diferença significativa entre a presença de resíduos faríngeos, penetração laríngea e aspiração laringotraqueal, independentemente da consistência avaliada, em comparação aos indivíduos disfágicos sem o diagnóstico. Em relação à gravidade dos resíduos faríngeos, o grupo com AVE apresentou maior frequência de resíduos de grau moderado a grave (YPRSRS 4-5), em comparação ao outro grupo, que apresentou maior frequência na gravidade de vestígio a resíduo leve (YPRSRS 2-3).

Sabe-se que os indivíduos com disfagia orofaríngea neurogênica apresentam resíduos faríngeos após a deglutição, pois essa alteração está relacionada com os distúrbios neuromusculares que envolvem o processo de eficiência da biomecânica da deglutição^(10-12)^. Porém, ainda são poucos estudos que comparam a presença e a gravidade dos resíduos faríngeos com outros indivíduos disfágicos sem o diagnóstico, em diferentes consistências alimentares^(14-16)^. Essas informações tornam-se importantes para compreender a fisiopatologia desses pacientes e o processo de reabilitação da função de deglutição, tendo em vista que há evidências que os resíduos faríngeos na disfagia pós-AVE são preditivos e persistentes durante toda sua recuperação^(17)^.

Com relação à avaliação fonoaudiológica prévia, mesmo que avaliado de forma subjetiva, a força e mobilidade de língua são medidas fundamentais para entender o processo de formação e transporte do bolo alimentar em cavidade oral. Por isso, há evidências que as lesões provenientes do AVE frequentemente resultam na fraqueza e descoordenação da língua, além de algumas relações com aspectos no processamento oral prejudicados^(18-20)^. Em nosso estudo, os resultados corroboram com a premissa da fraqueza lingual, visto que o grupo de disfágicos com AVE apresentou diferença significativa na mobilidade e força de língua, que em sua maioria estavam reduzidos, em comparação ao outro grupo. Assim, entende-se que os pacientes disfágicos pós-AVE desenvolvem maiores comprometimentos nos movimentos ântero-posteriores necessários para o transporte do bolo alimentar na cavidade oral, além da diminuição na força de retropropulsão desse alimento para a fase faríngea^(21,22)^. Isso ocorre devido os músculos intrínsecos da língua sofrerem redução da amplitude dos intervalos potenciais de ação motora, evidenciados pela abordagem eletrofisiológica^(23)^, o que resulta na diminuição dos movimentos da língua.

O resíduo faríngeo foi um achado importante no estudo, tendo em vista que houve diferença significativa na ocorrência e na gravidade dos resíduos faríngeos em todas as consistências alimentares entre os grupos. No grupo com AVE, houve maior ocorrência de resíduos na consistência do líquido extremamente espessado (nível 4) em grau moderado a grave, o que corrobora para a compreensão de que esses pacientes realizam deglutições ineficientes. Ademais, a classificação da gravidade dos resíduos nessa população ainda é divergente entre os estudos, visto que há evidências que relatam a presença de resíduos em grau leve^(14,24)^ e em outros em grau moderado a grave^(25,26)^. Essa diferença baseia-se principalmente nas características individuais da fase da doença e localização da lesão encefálica que o indivíduo sofreu, pois, há indícios de que lesões em áreas específicas do giro supramarginal, giro angular, giro pós-central e regiões corticais parieto-temporais possuem associações com maior presença de resíduos faríngeos^(26)^, o que indica áreas envolvidas na gravidade da disfagia nesses indivíduos.

Outro aspecto descrito na literatura relacionado aos resíduos faríngeos nessa população é a força faríngea, desencadeada pelo complexo hiolingual de elevação e abertura do esfíncter esofágico superior, descrita como fator preponderante sobre a ocorrência de resíduos faríngeos em valéculas e/ou seios piriformes^(27)^. Visto que, mesmo que haja ineficiência na abertura do esfíncter esofágico superior, essa dificuldade poderia ser superada pela pressão de retropropulsão do bolo alimentar pela língua e pela excursão hiolaríngea^(28)^.

Ao que se refere ao escape oral posterior, esse foi um achado videoendoscópico que não houve diferença significativa entre os grupos, apesar do grupo com AVE apresentar cerca de 56,8% de ocorrências na consistência de líquido ralo (nível 0). Essa característica é pouco descrita nas pesquisas que avaliam a função de deglutição pós-AVE, mas que são consequências dos mecanismos de atraso na resposta faríngea, assim como das dificuldades em mobilidade e força de língua para conter o bolo alimentar em cavidade oral, evidenciado na avaliação fonoaudiológica.

Com relação aos resultados de penetração laríngea e aspiração laringotraqueal, nota-se que houve diferença significativa entre os grupos, independente da consistência avaliada, na qual, foi observado que pacientes com disfagia pós-AVE apresentaram maior frequência dessas ocorrências, o que compromete o mecanismo de segurança da deglutição^(29)^. Na avaliação instrumental, há evidências de que esse achado relaciona-se diretamente com a gravidade dos resíduos após a deglutição, em que, a ocorrência de resíduos mais graves, seja em valéculas ou seios piriformes, contribuem para maiores chances de penetração e aspiração laringotraqueal nessa população, devido o déficit no gerenciamento de expulsão desse material na região laríngea^(30)^.

A tosse sob comando foi um dos parâmetros subjetivos avaliados antes do exame instrumental nesta pesquisa, na qual, não houve diferença entre os grupos. Apesar de não encontrar associação, esse parâmetro torna-se importante de ser pesquisado, visto que em alguns casos de AVE, poderá ocorrer sobreposição de lesões associadas a tosse fraca e até mesmo a diminuição da sensibilidade faríngea, o que piora o prognóstico de disfagia orofaríngea^(31)^. Embora os mecanismos de deglutição e tosse sejam independentes, ambas são ações motoras complexas e coordenadas que reconfiguram o padrão laríngeo do indivíduo^(31)^.

O tempo máximo de fonação é uma medida confiável para avaliação vocal, relacionada às medidas multimodais perceptivas e objetivas da gravidade da disfonia e, consequentemente, da gravidade da disfagia^(32,33)^. Em nosso estudo, houve diferença significativa entre os grupos, indicando que 59,1% dos indivíduos disfágicos pós-AVE em fase crônica tiveram o tempo máximo de fonação reduzido com referência ao seu gênero, o que sugere redução da coaptação das pregas vocais durante a fonação. Por ser um parâmetro rápido e fácil de ser coletado na prática clínica, confere ao fonoaudiólogo informações importantes quanto às possíveis alterações de fala e a proteção de vias aéreas inferiores durante a deglutição, além de ser um parâmetro pouco pesquisado nos pacientes disfágicos que aparentemente não possuem outras alterações na fala^(31)^. Visto que os resíduos faríngeos são frequentes, a coaptação glótica e o reflexo de tosse são funções que devem ser reabilitadas para desencadear com eficiência a reação de proteção de vias aéreas inferiores e a limpeza faríngea, de modo que evitasse as aspirações silenciosas que são comuns nos pacientes pós-AVE^(26)^.

Com relação às limitações do estudo, podemos citar o tipo do estudo não permitir causa e efeito, a causa idiopática de disfagia no grupo sem o AVE e que não foi possível diferenciar o mecanismo fisiológico da lesão, ou seja, a localização topográfica que prevaleceu no grupo com AVE. Sabe-se que a definição é importante para definir o prognóstico, reincidência e tratamento com medidas específicas, porém, é fato que todos os pacientes apresentavam diagnóstico de AVE do tipo isquêmico e em fase crônica da doença. Os resultados do presente estudo são capazes de fornecer uma caracterização objetiva e subjetiva das características da fisiopatologia envolvida na fase oral e faríngea nos pacientes disfágicos pós-AVE, em comparação aos pacientes disfágicos sem o comprometimento, de modo que seja possível levantar novas hipóteses com relação aos efeitos de novos programas terapêuticos na reabilitação fonoaudiológica.

## CONCLUSÃO

Os indivíduos disfágicos com diagnóstico de acidente vascular encefálico apresentaram diferença nos sinais videoendoscópios de resíduos faríngeos, penetração laríngea e aspiração laringotraqueal independentemente da consistência alimentar avaliada, em comparação aos indivíduos disfágicos sem o diagnóstico. Assim como houve diferença no achado da deglutição múltipla apenas nas consistências de líquido ralo, líquido extremamente espessado e sólido.
